# The struggle to make transparency mainstream: initial evidence for a slow uptake of open science practices in PhD theses

**DOI:** 10.1098/rsos.250826

**Published:** 2025-10-29

**Authors:** Hilmar Brohmer, Masia Fernanda Hoffmann

**Affiliations:** ^1^ Department of Psychology, University of Graz, Graz, Austria; ^2^ Psychology of Digitalisation, University of Bern, Bern, Switzerland; ^3^ Department of Psychology, Philipps University of Marburg, Marburg, Germany

**Keywords:** open science practices, open data, preregistration, early career researchers, behaviour change, meta science, research ethics

## Abstract

Open science (OS) practices—such as data sharing, study preregistration and transparent methods—aim to increase transparency of research. While OS practices are gaining popularity—particularly through bottom-up initiatives—their adoption rate among early career researchers remains unclear. To investigate this, we analysed dissertations from two German-speaking psychology departments with varying degree of OS implementation from 2018 to 2022. We manually coded *n* = 379 studies from *k* = 91 theses and surveyed former PhD students about perceived norms, attitudes and perceived behavioural control regarding OS practices. Our findings revealed a modest increase of OS over time but no significant difference between departments with more or less-established OS practices. Additionally, attitudes and perceived control appeared to affect OS use more than perceived norms of PhD students’ surroundings. As more than a decade has passed since the replication crisis emerged, this highlights a need to intensify measures at universities to implement OS.

## 1. Introduction

Open science (OS) practices, particularly data and materials sharing and study preregistration, have gained increasing attention among psychologists and social scientists over the last decade, as these disciplines were shaken by credibility and replication crises. Grassroots bottom-up OS initiatives, often led by early career researchers (ECRs) and methodologists, have played a significant role in promoting these practices [1–3]. Consequently, an increasing number of ECRs may have developed positive attitudes towards these practices and adopted them in their research. Here, we present—to our knowledge—the first study to combine coded data from PhD theses on the application of OS practices with survey data from former PhD students. We observe a gradual uptake of OS practices over time, as well as individual differences based on attitudes and perceived behavioural control.

### 1.1. Towards more open science

The credibility and replication crisis in psychology [4] emerged in the early 2010s amid scepticism over implausible findings (e.g. [5]) and a major replication study showing that fewer than 40% of studies in psychological research replicated [6]. Although concerns about low power and questionable research practices had long existed [7,8], the crisis underscored how common practices—like underpowered studies and selective reporting—systematically biased the literature [9]. While this called entire lines of research into question (e.g. self-control [10]; goal contagion [11]; social priming [12]), the distinct response came from methodologists and advocates of OS to promoted transparency and adherence to scientific norms [1,13,14], emphasizing that OS practices must be both accessible and rewarding to foster broad adoption of OS [15,16]. The goal of the newly emerging OS movement was a cultural shift aiming to enhance the transparency of research.

To increase transparency, three key OS practices have gained prominence. (i) The sharing of data and code, which is crucial for verifying reported results in research articles or reusing data for secondary analyses. (ii) The sharing of materials and methods, which enables independent replication of studies. (iii) The preregistration of studies, wherein researchers outline their sample sizes, analysis strategies and hypotheses before data collection to later distinguish confirmatory from exploratory results [17]. Several digital repositories have been developed to support these OS practices. In psychology, popular platforms include the Open Science Framework (OSF) by the Center for Open Science (https://osf.io), PsychArchives from the Leibniz Institute for Psychology (www.psycharchives.org) and ResearchBox by the Wharton Credibility Lab (along with the ‘AsPredicted’ preregistration form; https://aspredicted.org/researchbox_info.php), which provide the necessary infrastructure for all three OS practices.

Notably, OS practices have not only gained measurable popularity in recent years [18–20], but they have also demonstrated effectiveness: sharing data and materials has enhanced the analytic reproducibility of published research [21,22] and improved the replicability of studies by promoting more standardized research designs [23,24]. Importantly, study preregistration has led to the publication of smaller effect sizes and more null results [25], particularly in registered reports [26]. Preregistration has also enabled meta researchers to systematically detect deviations from preregistered plans [27] and assess the usefulness and specificity of different preregistration formats [28].

### 1.2. Open science among early career researchers

Despite the transparency benefits of OS practices, little systematic research exists on their adoption across psychological subfields (see [29]) and among ECRs, who are key to the future of academia. Journal data suggest that preregistration is used in about 40% of social and personality psychology articles [30], with similar findings in a survey of 700 psychologists, which also reported about 60% data sharing [31]. Notably, PhD students reported using OS practices 20% less than published authors, possibly due to selection effects or limited experience. This knowledge gap may also lead to less favourable attitudes towards OS [32]. More broadly, the effort required to implement OS practices may deter young researchers focused on producing publishable manuscripts.

ECRs are socialized into academia through their supervisors, colleagues and departments, learning the norms and behaviours valued for academic success [33,34]. Their perception of these norms likely influences their engagement with OS practices. Additionally, their own attitudes and confidence in applying OS—often shaped during their path to a PhD—also play a role. These factors—norms, attitudes and perceived behavioural control—are central to the theory of planned behaviour (TPB) [35,36], which has been widely used to explain behavioural outcomes.

### 1.3. The theory of planned behaviour and open science

As the adoption of OS can be seen as a large-scale behaviour-change challenge [37], this study applies the TPB framework to see how both environmental and personal factors shape OS engagement. Whether departments or individuals choose to adopt OS practices is not only a matter of behaviour but also hinges on the degree to which the actors are willing to embrace OS [38].

The TPB posits that an individual’s behaviour is influenced by their intention to perform the behaviour,^1^ which is shaped by three factors: (i) attitudes refer to the evaluation of a behaviour and its consequences as positive or negative; (ii) subjective norms encompass the social pressures individuals feel to engage in a behaviour based on the expectations of others; and (iii) perceived behavioural control involves an individual’s belief in their ability to successfully execute the behaviour.

Particularly behavioural control plays a crucial role in situations where behavioural decisions have to be made [35,36]. In the academic context, it may be that a PhD student is more likely to use OS practices in a study, when she or he feels that they can be applied successfully (e.g. data and materials can be transparently prepared and managed on a repository). The other elements are likewise important in this situation: supervisors and peers in the working environment of a PhD student provide environmental cues for normative guidance in situations, where it is unclear, which behaviour is appropriate (i.e. whether it is either ‘worth trying out OS practices’ or rather ‘a waste of time’). The student’s own attitude towards a beneficial behaviour can additionally affect decisions, even if behavioural control is low and norms are not present (as a colleague of the first author once said: ‘I will try to preregister my next study, even though I don’t know how it works yet and my supervisor does not know what it is’).

### 1.4. The present study

In this study, we investigated the uptake of OS practices among a relevant group of ECRs, namely, former PhD students. We compared studies conducted as part of dissertations from two large psychology departments in German-speaking countries. We considered a time effect (more OS practices over time) plausible as repositories like OSF became increasingly popular and several initiatives in favour of OS were started at universities throughout the last decade. However, recognizing differences in the speed and scope of the implementation of OS, norms may be more or less established at different departments regarding the number of researchers accepting and applying OS in general. Hence, we formulated the first hypothesis:

*H1*: OS practices are (a) increasingly used in studies by doctoral students between 2018 and 2022, and (b) more frequently in a department with more established OS practices as compared to a department with less established OS practices. Moreover, (c) the increase in OS practices over time is expected to be more pronounced in the department with less established OS practices than in the department with more established OS practices, as the latter is expected to have a higher baseline in 2018 (see expected pattern in electronic supplementary material, figure S1).

Beyond time and department effects, we were also interested in person-related factors—specifically, how PhD students perceived OS practices. We contacted former PhD students to ask about their perceived norms from supervisors, colleagues and departments, as well as their perceived behavioural control and attitudes towards OS practices at the time of the dissertation. Insights from these TPB measures can uncover the psychological mechanisms behind the adoption of OS practices. Hence, we formulated the second hypothesis:

*H2*: In addition to the H1 effects, (a) more positive attitudes towards OS practices, (b) higher perceived behavioural control, and perceived OS-friendly norms of (c) their supervisors, (d) their colleagues and (e) their department positively affect PhD students’ usage of OS practices in their dissertations.

## 2. Material and methods

### 2.1. Selected time period and psychology departments

Before developing a coding scheme, we decided to focus on a time period of 5 years between 2018 and 2022. We reasoned that PhD students, who submitted their PhD thesis during that time were likely exposed to the aftermath of the credibility and replication crisis while conducting their dissertation studies: They might have experienced the emergence of the OS movement (e.g. local OS initiatives at universities), were likely confronted with OS-related topics at major conferences, learned about major methodological changes in the field (e.g. in German-speaking countries [14,39]), or even followed heated OS-related debates on social media at the time (e.g. [40]). Hence, we considered it likely that PhD student had been exposed to and informed about the increasing importance of OS practices.

Moreover, we also selected two large psychology university departments (i.e. departments with a wide differentiation of psychology subfields), which could rely on local data management guidelines and, to our knowledge, had emphasized OS norms in the past—albeit to varying degrees and without fully mandatory implementation: The departments’ recent versions of data management guidelines were adopted in 2017 and 2019 at the two universities. However, to avoid identification of dissertation authors, we keep the department names masked. The selected departments were preliminarily distinguished as having either more established OS practices (i.e. researchers largely preregister studies and share their data and materials) or less established OS practices (researchers largely do not engage in these behaviours). To reaffirm this classification, we contacted representatives of the local OS initiatives and asked them to estimate the extent to which OS practices were applied by psychological researchers at their respective departments during the selected time period. In line with our assumptions, the representative of the former department indicated that most, if not all researchers likely engaged in these practices, whereas the representative of the latter department indicated that up to half of the researchers likely applied OS practices.

### 2.2. Coding scheme

We set up a detailed coding scheme (see complete original scheme at https://osf.io/he7rs/files/zsrj3) to capture extensive information on empirical studies conducted for dissertations in the two departments. This coding scheme contained—as primary information—the year of the dissertation being submitted and at which department it was submitted. For the dependent variable, we assessed whether a dissertation-related study (i) was preregistered, (ii) had openly accessible materials and (iii) had openly accessible data (each rated 1 = ‘yes’, 0 = ‘no’). To determine this, we applied various search terms to each dissertation. We used terms such as ‘OSF’, ‘PsychArch’ or ‘repos’ for references to common repositories, and combinations of ‘open’, ‘available’, or ‘accessible’ with ‘data’, ‘methods’, ‘code’ (which mostly comes together with open data) and ‘material’ to detect mentions of accessible materials and data. For preregistrations (or clinical trials), we searched for the terms ‘prereg’, ‘pre-reg’, ‘register’ or ‘trial’. We also manually checked data availability or OS statements, if applicable. As relevant criterium, studies needed to provide a working link to a repository (or supplemental documents when the article was openly accessible) for the OS practices, and we manually verified the functionality of these links. We also coded whether studies could or could not feasibly implement OS practices (e.g. when data were too sensitive to be shared openly). This information was independently coded by two coders (i.e. the second and the first author) and we assessed for interrater reliability (overall *κ* = 0.79 with 91% agreement) and discussed discrepancies [27]. The overall usability of this coding scheme was piloted by the second author on one PhD thesis before broader sampling began.

For descriptive purposes, we additionally coded the degree to which the three OS practices were provided: for data and materials, we coded whether the available information was minimal or comprehensive (e.g. with metadata, codebooks, detailed study materials, read me files, etc.); for preregistrations we checked, whether the available preregistration came in a short form (e.g. the AsPredicted template [41]) or in a long form (e.g. the OSF Preregistration template [28]). We also coded, if the dissertation study was part of an openly accessible published article, if the former PhD student was the first author of this article, and which psychological subfield the dissertation was part of. We also coded more information, including, e.g. whether a study used a repository to store data, the study’s sample size, whether it was conducted in the beginning, middle or end of the PhD. A list of all variables can be found online (https://osf.io/he7rs/files/bjk9m), and an overview of relevant variables is presented in table 1.

**Table 1.** Relevant variables from coding and questionnaire.

name	description	scale or labels
diss_id	id per dissertation (or PhD student)	id for mixed models
dep	department with more (‘more_OS’) or less established OS practices (‘less_OS’)	less_OS, more_OS
y_diss	year of dissertation submission and defense	years
TPB_att	during your dissertation studies, would you say it was important to you to use open-science practices? [attitudes towards OS]	7 = very important, 1 = not important at all
TPB_norm1	did your supervisor encourage you to use open-science practices?	7 = strongly encouraged, 1 = did not encourage
TPB_norm2	have colleagues in your department, with whom you often spent working hours, encouraged you to adopt open-science practices?	7 = strongly encouraged, 1 = did not encourage
TPB_norm3	has your department or university in general encouraged you to adopt open-science practices?	7 = strongly encouraged, 1 = did not encourage
TPB_pbc	would you say that it has been difficult for you to apply open-science practices in your dissertation due to various circumstances (e.g. time constraints in publishing, additional job, family, etc.)? [perceived behavioural control]	1 = very difficult, 7 = not at all difficult
OSpre	was the study preregistered on a repository (with a link)?	0 = no, 1 = yes
OSmat	does the study provide materials and methods (with a link to a repository or appendix, if article is OA)?	0 = no, 1 = yes
OSdat	does the study provide data (with a link to a repository or appendix, if article is OA)?	0 = no, 1 = yes
OS_practices	DV open science practices per study, sum of OSpre, OSmat, and OSdat, divided by the number of applicable pratices	0 = none, 1 = maximum
OS_rep	did the study mention a repository explicitely, where data, materials or the prereg was stored on? (link included)	0 = no, 1 = yes
st_sample_wins	reported study sample winsorized to a maximum of 500 and divided by 5	0 = zero participants, 5 = 500 participants
t_paper_re	time point/order of papers or within dissertation rescaled from 0 (first paper or study) to 1 (final paper or study); note that if only one study was conducted, this would receive 0.5	0 = first, 1 = final

### 2.3. Questionnaire

To gather additional information from the former PhD students, we sent out a questionnaire to them. As primary contact information was not always available in their dissertations (e.g. email addresses became invalid when they dropped out of academia), we also reached out via social networks (e.g. LinkedIn or Facebook) or contacted their previous supervisors to obtain updated contact information. Upon receiving functional email or social-network addresses, we decided to initiate contact up to three times. We formulated messages explaining the purpose of the study and included a link to the questionnaire, which contained an anonymizing code to later match responses with the coding scheme.

After providing their informed consent, participants completed the main part of the questionnaire, which consisted of eight questions. The first five questions were relevant for our analyses as they covered the TPB items (see table 1). Subsequently, we asked exploratory questions, which can be found in the document in the electronic supplementary material. They were on participants’ university, where they received the master’s degree and whether they had already learned about OS practices at that university. Finally, participants were asked to indicate whether they felt they focused exclusively on their dissertation during their PhD or whether they were also actively engaged in academic activities aimed at advancing the field.

### 2.4. Design and power

To test H1, we preregistered this study with a 5 (between; time: 2018–2022) × 2 (between; department: less vs. more established OS) design for a general linear model. Additionally, we accounted for the nested structure of the data, as we expected PhD students to have multiple studies (Level 1) nested within their dissertations (Level 2). For H2, we preregistered multilevel models that included TPB predictors, extending the model from H1. The dependent variable—the percentage of OS practices per study, accounting for the feasibility of each OS practice—was determined beforehand.

We estimated a realistic sample size in advance using the smaller of the two university departments as a benchmark, where we had preliminary identified an average of six dissertations per year. Each dissertation, we assumed, likely contains multiple studies, typically embedded in around three published papers or book chapters, as required by the curriculum. Based on this, we conservatively estimated an average of two studies per paper or chapter, assuming that some psychological subfields (e.g. social psychology) often report several studies per paper, while others (e.g. neuropsychology) may focus on a single study. This led to an estimated total sample size of *n* = 360 studies (*n* = 180 per department).

A sensitivity power analysis revealed that this sample size would be sufficient to detect main and interaction effects in a linear model of *η_p_*² = 0.03 (based on *f* = 0.18, power = 80%, α-error rate = 5%, numerator *df* = 4; calculated in G*Power, version 3 [42]). This corresponds to a small to medium effect size, based on empirically derived effect size estimates [43]. For the main effect of department (H1b), this effect size would be equal to *d* = 0.36 [44], our equivalence threshold. The effect of time was expected to follow a linear trend (using a polynomial contrast; H1a), similar to standardized coefficients of *β* = 0.13. We also used this estimate as benchmark for models including the TPB predictors.

### 2.5. Sampling procedure

**Figure 1.**
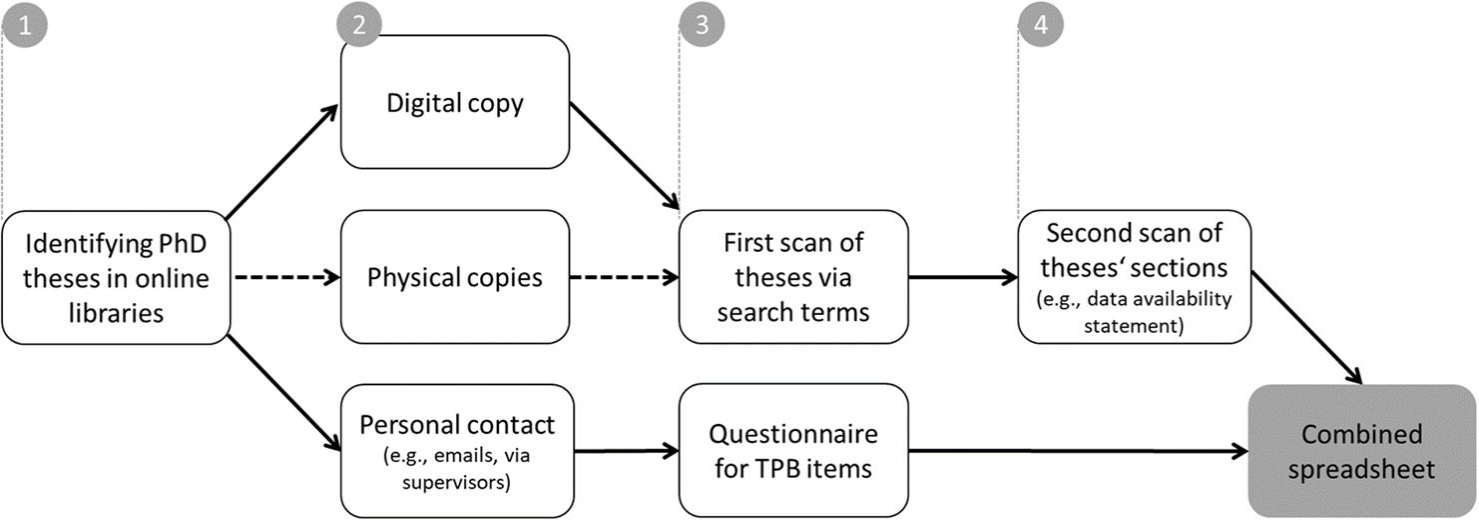
Procedure of the study in four steps.

We followed the procedure outlined above and in figure 1. We first identified relevant dissertations on the university systems and online libraries. We then obtained digital copies of the dissertations from the online libraries if available, or requested digital copies from the original authors (or their supervisors), or obtained a hard copy from the library (if no digital copy was available). However, for the department with less-established OS norms—which we coded first—the total number of studies per dissertation was lower than anticipated: instead of 180 studies, we identified 121 studies, of which four were excluded from coding because they were either qualitative use case studies without samples (*n* = 3) or could not be obtained from the library or from the first author or supervisor after repeated contact attempts (*n* = 1). Hence, we coded the remaining *n* = 117 studies (*k* = 29 PhD theses) from this department.

In contrast, for the department with more-established OS practices, we encountered the opposite issue: more dissertations—partially twice as many per year—were available than anticipated. To stay close to our preregistered sample size of *n* = 360 studies and account for the possibility that some studies might not be eligible for coding (as it was the case in the other department), we decided to randomly select ten to eleven dissertations per year from this department. This process yielded *n* = 268 studies, of which five were excluded (qualitative use cases: *n* = 1; software descriptions: *n* = 3; study protocol without study: *n* = 1). This left us with *n* = 263 studies (*k* = 62 PhD theses) from the second department, resulting in a total sample of *n* = 379 studies. Notably, for H2, the sample size was smaller (*n* = 247, *k* = 59), as not all PhD students responded to our request to participate in the survey.

## 3. Results

Upon completing the coding of the dissertations, our sample consisted of *n* = 379 studies from *k* = 91 dissertations. Most studies came from cognitive (*n* = 99), social (*n* = 75) or clinical psychology (*n* = 75). Fewer studies were identified in neuro and biological (*n* = 46) and health or medical psychology (*n* = 30), and only a fraction came from pedagogical (*n* = 26), developmental (*n* = 13), meta psychology and statistics (*n* = 15). A relative majority of PhD students (*k* = 36) did not apply any OS practices in their dissertations. Some applied OS practices in up to 25% of their studies (*k* = 27), up to 50% (*k* = 19), and only a small fraction used OS practices in more than 50% of their studies (*k* = 9). In 2018, most studies did not apply any OS practices (74%, 57/77 studies), whereas in 2022, it was only a minority (41%, 40/98 studies). We conducted our analyses in *jamovi* [45] and *R* [46] using the *GAMLj* package [47].

### 3.1. Test of hypothesis H1

#### 3.1.1. Preregistered

**Figure 2.**
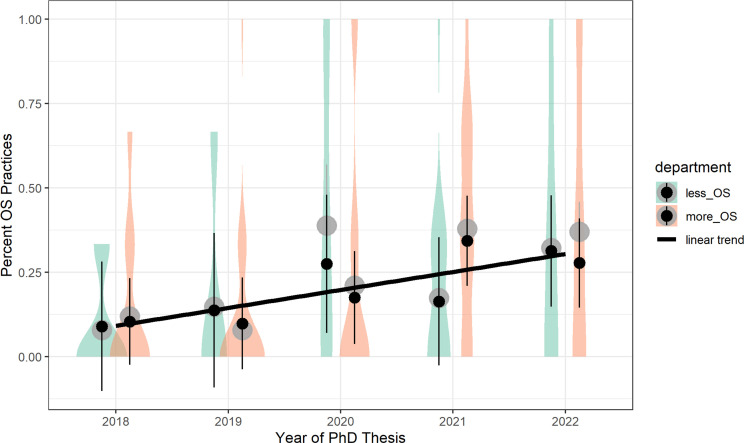
Main results for H1. Note: larger grey circles represent means from the general linear model (registered); small black circles with whiskers represent means and 95% CIs from the linear mixed model (non-registered); linear trend is based on the time main effect; violins represent distribution of the data per year and department. Plot was created using the *ggplot2* package [48].

We performed a linear model with the percentage of OS practices per study as the dependent variable, and time and department as categorical predictors, including their interaction, in a 5 (between, time: 2018–22)×2 (between, department: less vs. more established OS norm) design. We found a main effect for time, *F*(4, 369) = 3.23, *p* < 0.001, *η_p_*² = 0.085: As hypothesized in H1a, this main effect followed a linear polynomial contrast for time, *t*(369) = 5.54, *p* < 0.001, *b* = 0.208, s.e. = 0.035, *β* = 0.635. Contrary to H1b, there was no significant effect of department, *F*(1, 369) = 0.07, *p* = 0.799, *η_p_*² < 0.001, *β* = 0.027. This effect was equivalent to zero with respect to the preregistered threshold ∆*d* ± 0.36, *d* = 0.02, 90% CI [−0.16; 0.22]. Moreover, there was an interaction effect, *F*(4, 369) = 3.41, *p* = 0.009, η*_p_*² < 0.036, but the pattern did not confirm H1c: the departments did not differ meaningfully in 2018 and 2019, showed a cross-over pattern in 2020 and 2021, and then converged again in 2022 (see figure 2; see electronic supplementary material, table S1 for model statistics, and table S3 for simple effects).

#### 3.1.2. Non-registered

As a robustness check, we performed additional non-registered tests of H1a–c. We found support for H1a, but not for H1b or H1c. First, we nested studies within dissertations in a mixed model with random intercepts (*R*² conditional = 0.51, *R*² marginal = 0.09, *ICC* intercept = 0.46; figure 2, electronic supplementary material, table S2). Again, we found a significant polynomial contrast for time, *t*(87.91) = 2.95, *p* = 0.004, *b* = 0.168, s.e. = 0.057, *β* = 0.514 (see figure 2, electronic supplementary material, table S2 for model statistics, electronic supplementary material, table S4 for simple effects). A similar pattern emerged when treating the dependent variable as ordinal and zero-inflated (i.e. Poisson distributed) in a generalized mixed model: the polynomial time trend remained the only significant effect, *z* = 3.41, *p* < .001, ln(*OR*) = 1.623, s.e. = 0.476 (see electronic supplementary material, table S5).

### 3.2. Test of hypothesis H2

As preregistered, we tested the effect of the TPB items in a follow-up multilevel model with random intercepts for studies. Using this reduced sample (*n* = 247), we first included the variables from H1a–c and then added the TPB items. This model (see table 2) explained a statistically significant proportion of variance, *R*² conditional = 0.58, *R*² marginal = 0.33, *ICC* intercept = 0.38. Only participants' perceived behaviour control (H2b), *t*(46.49) = 2.91, *p* = 0.006, *b* = 0.068, s.e. = 0.023, *β* = 0.207, and positive attitudes (H2a) towards OS, *t*(44.90) = 2.37, *p* = 0.022, *b* = 0.069, s.e. = 0.029, *β* = 0.209, positively predicted the use of OS practices in dissertations. Meanwhile, the time effect (H1a) was reduced, *t*(41.60) = 1.94, *p* = 0.059, *b* = 0.137, s.e. = 0.071, *β* = 0.419. Norms from supervisors, colleagues, or departments (H2c–e) did not significantly contribute to the model, *β* ≤ 0.073, *p* ≥ 0.401.

**Table 2.** Fixed effects parameters of the test for H2. Note. DV is OS practices (continuous scale 0–1); conditional *R*² = 0.583; marginal *R*² = 0.332; ICC (random intercepts diss_id) = 0.376; DF method: Scatterwaite; CI method: Wald; model information: lmer model with bobyqa optimizer; all predictors are centred around the mean.

variable	*b*	s.e.	*β*	***β*** **LB 95% CI**	***β*** **UB 95% CI**	*df (t)*	*t*	*p* (*t*)	num df (*F*)	den df (*F*)	*F*	*p* (*F*)
(intercept)	0.276	0.030	0.091	−0.091	0.272	40.827	9.133	< 0.001				
y_diss linear	0.137	0.071	0.419	−0.007	0.844	41.602	1.940	0.059^a^	4	42.997	1.046	0.395
y_diss quadratic	0.023	0.067	0.069	−0.332	0.470	42.432	0.339	0.736				
y_diss cubic	−0.013	0.069	−0.039	−0.455	0.377	44.488	−0.186	0.853				
y_diss quartic	0.048	0.063	0.147	−0.233	0.528	43.640	0.763	0.450				
dep: more_OS vs. less_OS	−0.113	0.070	−0.344	−0.767	0.078	43.015	−1.605	0.116	1	43.015	2.575	0.116
TPB_norm1 - supervisor	−0.014	0.034	−0.043	−0.246	0.159	45.395	−0.423	0.674	1	45.395	0.179	0.674
TPB_norm2 - colleagues	0.024	0.028	0.073	−0.096	0.242	42.549	0.849	0.401	1	42.549	0.721	0.401
TPB_norm3 - department	0.001	0.032	0.002	−0.192	0.197	45.063	0.022	0.983	1	45.063	0.000	0.983
TPB_pbc	0.068	0.023	0.207	0.067	0.347	46.487	2.910	0.006^b^	1	46.487	8.469	0.006
TPB_att	0.069	0.029	0.209	0.035	0.383	44.897	2.372	0.022^b^	1	44.897	5.628	0.022
y_diss × dep linear	0.153	0.142	0.468	−0.385	1.32	41.715	1.081	0.286	4	42.528	1.144	0.349
y_diss × dep quadratic	−0.168	0.126	−0.514	−1.275	0.247	42.686	−1.331	0.190				
y_diss × dep cubic	−0.149	0.135	−0.454	−1.265	0.357	42.598	−1.103	0.276				
y_diss × dep quartic	0.01	0.128	0.031	−0.74	0.802	42.615	0.079	0.937				

^a^ The linear time trend is not statistically significant (*p* = 0.059), but above the preregistered equivalence threshold of *β *> 0.19.

^b^ Statistically significant effects (*p* < 0.05) above the preregistered equivalence threshold of *β* > 0.19.

### 3.3. Exploratory analyses

In the academic context, norms of the environment can be seen as crucial in shaping PhD students own attitudes and perceived behaviour control. As we argued above, supervisors, colleagues or the department may highlight OS as integral part of academia and enable students to actually engage in OS practices. Hence, this chain of variables can be seen as mediation. We tested this mediation with the three norms predicting PhD students’ attitudes and behaviour control, which in turn predict OS practices using *PATHj* [49]. We only found evidence that the supervisor’s norm was associated with behavioural control, *z* = 4.75, *p* < 0.001, *b* = 0.749, s.e. = 0.158, *β* = 0.729, which in turn predicted OS practices, *z* = 2.36, *p* = 0.019, *b* = 0.046, s.e. = 0.019, *β* = 0.296. The indirect effect was statistically significantly different from zero, *z* = 2.11, *p* = 0.035, *b* = 0.034, s.e. = 0.016, *β* = 0.216, but should be interpreted with caution due to our small sample size (see details in the document in the electronic supplementary material).

**Figure 3.**
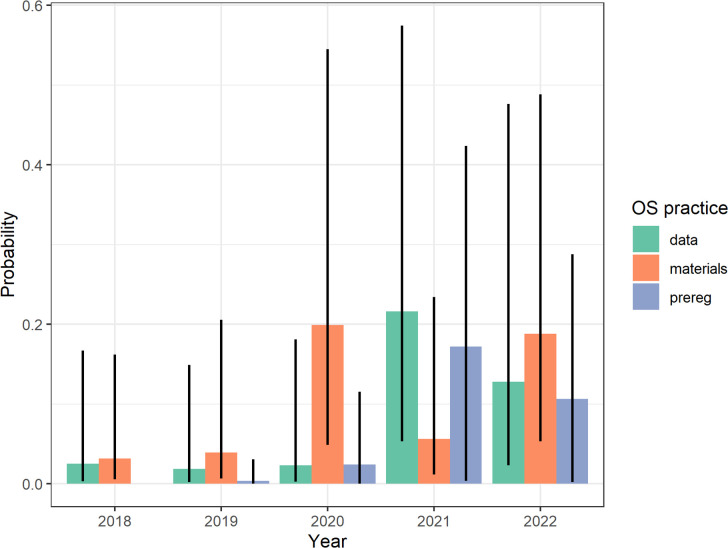
Exploratory results for individual OS practices over time. Probability estimates are taken from generalized logistic mixed models; vertical lines represent 95% CIs.

We collected additional data on the PhD theses, including several study-level information, which interested readers can access online. Here, we focus on two analyses. First, we examined whether the use of OS practices increased over time across all three domains—open data, materials and preregistration—or whether there were differences between them. To do so, we ran three logistic mixed models with random intercepts for studies, using time as independent variable. As shown in figure 3, PhD students largely did not engage in any practices in 2018 and 2019 (each practice, *P* < 0.05), but then, practices gained more traction. By the final year 2022, materials sharing was the most frequent practice (*P* = 0.19), followed by data sharing (*P* = 0.13)^2^ and preregistration (*P* = 0.11). However, only data sharing showed a significant polynomial linear increase over time, *z* = 2.18, *p* = 0.029, ln(*OR*) = 1.959, s.e. = 0.899.

Second, we explored three variables that—despite not necessarily causally predicting OS uptake—might be associated with increased OS adoption: (i) greater use of online repositories, (ii) larger sample sizes in experimental studies due to *a priori* planning, and (iii) increased awareness and proficiency over time within a dissertation project (i.e. an individual person time effect). We tested these variables in linear mixed models similar to H2. All three variables were significantly associated with a greater use of OS practices (repository use (0 = no, 1 = yes): *b* = 0.51, s.e. = 0.03, *p* < 0.001; sample size in experiments (1 = 100 participants to 5 = 500 and more participants): *b* = 0.036, s.e. = 0.02, *p* = 0.025; studies (0 = first, 1 = last): *b* = 0.06, s.e. = 0.03, *p* = 0.048).

## 4. Discussion

To our knowledge, we conducted the first systematic study of OS practices and psychological factors by combining coded data from dissertations with survey data from former PhD students. Our primary findings suggest a modest linear trend towards increased use of OS practices in PhD theses over time, indicating that such practices are indeed becoming more popular among ECRs. After all, a majority of the sampled studies (disregarding the PhD thesis level) contained a least one OS practice in 2022. Although the increase from an average of approximately 10% OS practice adoption in 2018 to 36% in 2022 appears substantial, it also indicates that a large majority of ECRs still does not use OS practices in their dissertation-related studies. Extrapolating this trend suggests that a widespread adoption—exceeding 90%—may be reached in the 2030s, provided the linear trend continues without plateauing. Since the trend began from a near floor level in 2018, with few to no OS practices observed, and evolved into a bimodal distribution by 2022, it appears that some PhD students now apply OS practices extensively, while others continue to abstain from using them.

Interestingly, we did not observe the hypothesized department effect, nor the expected interaction effect. As our analysis relied on estimates of perceived implementation provided by local OS initiatives, this suggests that some initiatives may overestimate their actual impact and the extent of cultural change already achieved. This overestimation may stem from a bubble bias among OS proponents: having engaged a considerable number of ECRs to adopt OS, proponents may develop the illusion that these norms are widely adopted—even though a substantial portion of ECRs may never attend their events.

Psychological differences among ECRs—in line with the TPB—also appear to account for some of the variation in the use of OS practices. ECRs who held more positive attitudes towards OS practices or believed they had sufficient time and resources to implement them were, in fact, more likely to do so. Surprisingly, the perceived norms communicated by ECRs’ immediate surroundings, such as supervisors, colleagues or departments, did not affect their practices—at least not directly. This was contrary to our hypotheses, as we did expect effects of all TPB variables. Hence, we reason that norms of the environment possibly do not directly affect PhD students behaviour when it comes to conducting studies.

But as suggested by our exploratory findings, the supervisors may exert their influence rather indirectly, as they may communicate their norms in favour (or against) OS practices. Thereby they potentially encourage their PhD students to actually devote some time to engage in OS practices, as explained by the perceived-control path. After all, PhD students eventually conduct their research relatively independently, a skill that they need to learn during the time of their PhD—their supervisors can rather provide guidance of how to do it ‘correctly’.

However, we emphasize that these findings—particularly those concerning individual differences—should be considered preliminary. Despite multiple attempts to contact former PhD students, not everyone participated in the survey. Our limited sample has reduced statistical power [50] and may also introduce selection bias, meaning that the observed effects may not generalize to non-participating ECRs. Furthermore, as the TPB-related questions were asked several years after participants completed their PhDs, it is possible that some respondents did not accurately recall their earlier attitudes, perceived norms, or behavioural control regarding OS practices. A more informative approach would certainly be to track current PhD students on their individual-difference measures during their PhD studies.

Another limitation is that the two Germanophone psychology departments, from which we sampled the PhD theses, were selected based on two considerations: first, their relatively large number in sections provided us with a broad scope of topics, covering psychology as whole; second, the rate of implemented OS practices at departments—or lack thereof—we got communicated from local OS initiatives, was relevant for the main hypotheses for cultural differences between departments. However, as this was a non-random selection, our findings cannot be directly generalized to other psychology departments. For example, in smaller departments with fewer sections, it may be easier to implement OS practices more swiftly and effectively. Additionally, substantial cultural differences may exist not only between departments but also across countries. In Germany and the United Kingdom, for instance, major psychological associations addressed the credibility and reproducibility crisis relatively early. In contrast, other countries may have responded later, potentially leading to lower uptake of OS practices due to a lack of widespread awareness.

Moreover, another potential limitation of the present study is the possibility of restricted variance due to the selection of departments: Both departments investigated had established local OS initiatives, which may indicate a higher-than-expected baseline level of awareness and engagement compared to departments without such initiatives. This selection could have unintentionally narrowed the range of variability in OS uptake, causing our dependent variable to lack sufficient contrast to detect meaningful variation (as it was potentially the case for the final year, see figure 2, year 2022). Future research might address this limitation by including departments with more diverse levels of OS infrastructure and culture to better capture the full spectrum of uptake.

Finally, as we argued in the introduction, we selected PhD theses as a proxy for research conducted by ECRs. This choice also means that our findings cannot be readily generalized to later career stages. Senior postdoctoral researchers and professors at the time may have had fewer incentives to change their research practices, particularly if they were already publishing successfully before the credibility crisis emerged in psychology. Some may have adhered even more strongly to the status quo and might only be swayed by top-down policy interventions [1].

Our findings should be regarded as a starting point for larger, systematic studies aimed at comparing the uptake of OS practices over time, particularly since the onset of the credibility and replication crisis around 2011/2012. To better understand how and when these practices became popular among ECRs, it will be essential to examine a broader range of PhD theses from different departments, thereby increasing the statistical power of the analyses. Furthermore, future studies could incorporate additional key factors, such as PhD students’ prior exposure to OS-related content during their undergraduate education or whether their departments' doctoral curricula included mandatory courses on OS practices. On a broader scale, such research could inform whether the efforts of grassroots OS initiatives should be more systematically complemented by top-down policy measures from universities or funding bodies—for instance, through initiatives like CoARA for responsible research assessment [51]—especially if uptake levels show signs of plateauing.

## 5. Conclusion

In this study, we investigated the uptake of OS practices in PhD theses from two psychology departments, along with psychological predictors of their use. We found that while OS practices have become more common over time in both departments, overall uptake remains low, highlighting the need for stronger top-down measures to motivate ECRs to adopt OS. Higher perceived behavioural control and more positive attitudes towards OS among PhD students were associated with greater OS uptake, and the perceived norms of supervisors may have influenced students’ behavioural control. Further research examining PhD theses across additional departments and countries is needed to determine whether these patterns generalize.

## Data Availability

This study applied Open Science practices: All materials (including coding scheme and variable list) are available from https://osf.io/he7rs/overview. The data and analysis codes are also available from https://osf.io/he7rs/overview. The study was preregistered at https://osf.io/zghne. The supplementary material, where we report minor deviations from the preregistration, are available from https://osf.io/he7rs/files/g48jz. Supplementary material is available online [52].

## Footnotes

^1^
Behavioural intention is an intermediate step in the TPB framework and important when actual behaviours are hard to measure empirically. Luckily, as we look at actual behaviour (i.e. uptake of OS) directly, we focus on the other elements of the TPB.

^2^
As a reviewer noted, sharing of analysis code is likewise important for reproducibility. As we also gathered information on analysis code separately, we checked its frequency: of all *n* = 100 studies that provided a link to open data, *n* = 77 also provided a link to analysis code. There was not a study that provided code despite not having open data.
